# Simultaneous 18F-FDG PET/MRI Radiomics and Machine Learning Analysis of the Primary Breast Tumor for the Preoperative Prediction of Axillary Lymph Node Status in Breast Cancer

**DOI:** 10.3390/cancers15205088

**Published:** 2023-10-21

**Authors:** Valeria Romeo, Panagiotis Kapetas, Paola Clauser, Sazan Rasul, Renato Cuocolo, Martina Caruso, Thomas H. Helbich, Pascal A. T. Baltzer, Katja Pinker

**Affiliations:** 1 Department of Advanced Biomedical Sciences, University of Naples Federico II, Via S. Pansini 5, 80138 Naples, Italy; valeria.romeo@unina.it (V.R.); martina.caruso@unina.it (M.C.); 2 Department of Biomedical Imaging and Image-Guided Therapy, Division of General and Pediatric Radiology, Medical University of Vienna, Waehringer Guertel 18-20, 1090 Wien, Austria; 3 Department of Biomedical Imaging and Image-Guided Therapy, Division of Nuclear Medicine, Medical University of Vienna, Waehringer Guertel 18-20, 1090 Wien, Austria; sazan.rasul@meduniwien.ac.at; 4 Department of Medicine, Surgery, and Dentistry, University of Salerno, 84081 Baronissi, Italy; renato.cuocolo@unisa.it; 5 Augmented Reality for Health Monitoring Laboratory (ARHeMLab), Department of Electrical Engineering and Information Technology, University of Naples “Federico II”, 80131 Naples, Italy; 6 Department of Biomedical Imaging and Image-guided Therapy, Division of Structural Preclinical Imaging, Medical University of Vienna, Waehringer Guertel 18-20, 1090 Wien, Austria; 7 Department of Radiology, Breast Imaging Service, Memorial Sloan Kettering Cancer Center, 300 E 66th Street, New York, NY 10065, USA

**Keywords:** breast cancer, positron emission tomography, magnetic resonance imaging

## Abstract

**Simple Summary:**

A Machine Learning-based radiomics approach applied to hybrid 18F-FDG PET/MRI might predict axillary lymph node involvement in breast cancer based on the analysis of primary cancer lesions. Indeed, the depiction of tumor heterogeneity through the extraction of radiomics features for both morphological and functional images can be representative of tumor aggressiveness and propensity to invasion. In light of the new personalized treatment options, such as neoadjuvant systemic treatments or the possibility of omitting sentinel lymph node biopsy in patients with a clinically negative axilla, such an approach could result in a better preoperative stratification and also make hybrid 18F-FDG PET/MRI a comprehensive tool for both local and node (N) staging in breast cancer.

**Abstract:**

In this prospective study, 117 female patients (mean age = 53 years) with 127 histologically proven breast cancer lesions (lymph node (LN) positive = 85, LN negative = 42) underwent simultaneous 18F-FDG PET/MRI of the breast. Quantitative parameters were calculated from dynamic contrast-enhanced (DCE) imaging (tumor Mean Transit Time, Volume Distribution, Plasma Flow), diffusion-weighted imaging (DWI) (tumor ADCmean), and PET (tumor SUVmax, mean and minimum, SUVmean of ipsilateral breast parenchyma). Manual whole-lesion segmentation was also performed on DCE, T2-weighted, DWI, and PET images, and radiomic features were extracted. The dataset was divided into a training (70%) and a test set (30%). Multi-step feature selection was performed, and a support vector machine classifier was trained and tested for predicting axillary LN status. 13 radiomic features from DCE, DWI, T2-weighted, and PET images were selected for model building. The classifier obtained an accuracy of 79.8 (AUC = 0.798) in the training set and 78.6% (AUC = 0.839), with sensitivity and specificity of 67.9% and 100%, respectively, in the test set. A machine learning-based radiomics model comprising 18F-FDG PET/MRI radiomic features extracted from the primary breast cancer lesions allows high accuracy in non-invasive identification of axillary LN metastasis.

## 1. Introduction

Multimodal functional imaging allows the depiction of hallmarks of cancer, such as neoangiogenesis and the activation of invasion and metastasis, as well as the prediction of response to treatment based on the calculation of quantitative parameters [1,2,3]. Further, advances in software and hardware development allow the possibility of exploring image heterogeneity in terms of pixel distribution and quantifying it through the extraction of numeric data [4,5]. This process, defined as radiomics, can provide a large amount of data that can be processed by artificial intelligence (AI) approaches, such as machine and deep learning, to develop predictive models [6].

Breast cancer is the most common solid malignancy affecting women [7]. Accurate preoperative assessment of axillary status is mandatory to determine the most appropriate treatment strategy [8]. In clinical practice, various imaging modalities are used to identify suspicious lymph nodes, with variable diagnostic performance and sensitivity and specificity ranging from 64–87% and 40–100% for malignancy [9]. Indeed, in most cases, the final diagnosis of malignant lymph nodes is based on histology after surgical excision through sentinel lymph node biopsy or axillary dissection. Given the currently debated “axillary de-escalation approach,” such invasive procedures could be avoided in patients with no or only mild nodal involvement [10]. Particularly, several clinical trials are investigating the possibility of omitting sentinel lymph node biopsy in a clinically negative axilla [11].

AI approaches have been applied to MRI and 18F-FDG PET/CT to predict breast cancer outcomes, including axillary lymph node status, even with some preliminary clinical applications [12,13,14]. Recently, simultaneous PET/MRI systems have been introduced, opening up new avenues of research focused on developing predictive AI models based on comprehensive functional tumor properties on imaging [15,16]. We hypothesized that radiomics analysis of simultaneously acquired functional and metabolic imaging data from MRI (including diffusion and perfusion sequences) and PET could capture tumor heterogeneity associated with tumor aggressiveness and spread, consequently enabling non-invasive prediction of axillary lymph node metastasis. To test this hypothesis, we built a radiomics-based machine learning (ML) model to predict the presence of axillary lymph node metastasis in patients with breast cancer using radiomic features and/or quantitative parameters extracted from simultaneous 18F-FDG PET/MRI.

## 2. Materials and Methods

### 2.1. Patient Sample

**Figure 1:**
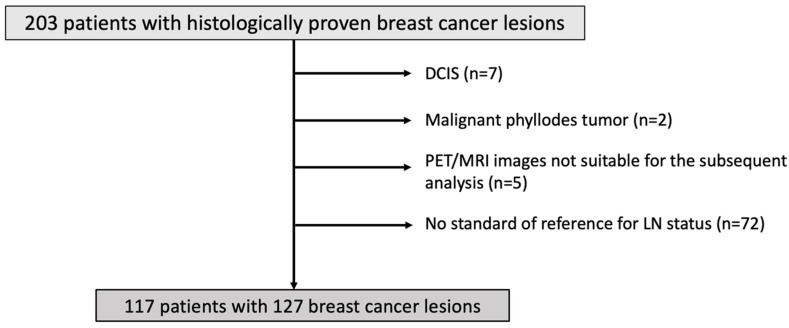
Flowchart of patient inclusion.

The local Ethics Committee approved this prospective, single-institution study, and all subjects obtained written informed consent. Adult patients (>18 years old) with suspicious or histologically proven breast tumors (BI-RADS 4 to 6) who were not pregnant and not breastfeeding were enrolled and underwent simultaneous 18F-FDG PET/MRI of the breast between June 2016 and June 2022. The exclusion criteria were: contraindications to contrast-enhanced MRI (e.g., impaired renal function); known biopsy-proven cancer with a low probability of lymph node metastasis (e.g., ductal carcinoma in situ or malignant phyllodes tumor); lack of an appropriate standard of reference for axillary lymph node; and images not suitable for the subsequent analysis (e.g., DWI images significantly affected by artifacts, DCE-MRI sequences incomplete and thus not suitable for the calculation of quantitative perfusion parameters). The flowchart of patient inclusion is given in Figure 1.

The final study sample comprised 117 female patients (mean age: 53 years, age range: 25–82 years). Of these, 85 and 72 patients have been previously reported in two prior articles dealing with the prediction of breast cancer diagnosis and molecular subtypes, respectively [17,18].

### 2.2. Reference Standard

The reference standard for axillary lymph node status was preoperative ultrasound-guided lymph node biopsy and/or sentinel lymph node biopsy or axillary dissection. Axillary status was also considered negative if the patient was a candidate of neoadjuvant chemotherapy with no evidence of lymph node malignancy before and after treatment, as demonstrated by sentinel lymph node biopsy. Similarly, axillary status was also considered positive if the patient was a candidate of neoadjuvant chemotherapy with clear evidence of lymph node malignancy before and after treatment, as demonstrated by sentinel lymph node biopsy.

### 2.3. 18F-FDG PET/MRI Examination

Simultaneous 18F-FDG PET/MRI was acquired using a hybrid PET/MRI system (Biograph mMR system, Siemens, Germany), which consisted of an MRI-compatible PET detector embedded in a 3T MRI scanner [17]. The imaging examination began 60 min after the 18F-FDG injection. MRI consisted of T2-weighted, diffusion-weighted imaging (DWI), and dynamic contrast-enhanced (DCE) sequences with high temporal resolution, performed before and after the injection of a gadolinium-based paramagnetic contrast agent (Dotarem: 0.2 mL/kg body weight). The imaging protocol, including MRI acquisition parameters, is detailed in Supplementary Material S1.

### 2.4. Quantitative Parameters Analysis

A dedicated breast radiologist (**) with 7 years of experience and a nuclear medicine physician (**) with 11 years of experience independently assessed MRI (T2-weighted, DWI, DCE) and PET images, respectively. DWI images were first assessed. According to the recent European Society of Breast Imaging recommendations, circle regions of interest (ROIs) were placed at the level of darkest signal intensity areas, using T2-weighted and DCE images as references to exclude areas of necrosis [19], and ADCmean was measured. In addition, ADCmean of the ipsilateral breast parenchyma was also measured. DCE images were imported into a freely available software (Horos v3.3.6), and a fast-deconvolution method was applied using a freely available Osirix plugin compatible with Horos to calculate mean transit time (MTT), volume of distribution (VD), and plasma flow (PF) perfusion parameters [20,21]. Details on perfusion parameters measurements are reported in Supplementary Material S2. Whole-tumor uptake of 18F-FDG, in terms of maximum, mean, and minimum standardized uptake values (SUVmax, SUVmean, and SUVmin), was calculated on PET images avoiding the inclusion of background parenchymal uptake. A threshold of 90% of SUVmax corrected for local background was used. Parenchymal uptake (SUVmean) of the ipsilateral breast tissue was also measured, keeping an appropriate distance from the nipple-areolar complex. PET assessment was conducted using a Hermes Hybrid Viewer (Hermes Medical Solutions, Stockholm, Sweden). This method proved to be reproducible in previous experiences [17,18]

### 2.5. Tumor Segmentation

Whole lesions were segmented on MRI (T2-weighted, DWI, DCE) and PET images using freely available software (ITK-SNAP v3.6.0). Semi-automated segmentation was used for DWI, DCE, and PET images by exploiting the high contrast between tumor lesions and the surrounding breast parenchyma. In contrast, manual segmentation was used for T2-weighted images, using a slice-per-slice approach. In all cases, necrotic and cystic areas and surrounding/intralesional blood vessels were excluded from volumes of interest. Volumes of interest and their corresponding whole-breast images were coupled to perform radiomic feature extraction. Apparent diffusion coefficient (ADC) maps were used for feature extraction based on the volumes of interest on DWI images.

### 2.6. Image Pre-Processing

Open-source Python-based software (PyRadiomics, v3.0.1) was used for image pre-processing and 3D radiomics feature extraction in compliance with the Image Biomarker Standardization Initiative [22]. To ensure rotational invariance of textural features, pixel resampling was performed with the spacing set to 1 × 1 × 1 mm [23,24]. Gray-level whole-image normalization was paired with a scaling of 100 and a voxel array shift of 300, resulting in a range of 0–600. A fixed bin width of 64 was used for grey-level discretization. Wavelet and Laplacian of Gaussian (log) filters (sigma = 1, 2, 3) were also applied to enhance image textural properties.

### 2.7. Radiomics Features Extraction and Selection

The patient sample was divided into a training (70%) and a test (30%) set. The following classes of radiomic features were extracted from both non-filtered and filtered images: 3D shape, first order, gray level cooccurrence matrix, gray level run length matrix, gray level size zone matrix, gray level dependence, and neighboring gray-tone difference matrix. According to PyRadiomics recommendations, all available features were calculated except for the gray level cooccurrence matrix sum average. Indeed, this feature is considered redundant with other gray-level cooccurrence matrix parameters. A Simple Imputer based on mean values was fitted on the training set data and used to substitute missing non-radiomic features (i.e., quantitative DWI and/or perfusion parameters) in the training and test sets. To avoid any information leakage, a MinMax scaler with a range of 0–1 was fitted only on the training data and then used to transform training and test sets. Then, multi-step feature selection was performed. First, non-informative features showing low variance (≤0.01) were excluded. Thereafter, features with high collinearity were discarded based on the results of the pairwise correlation matrix (Pearson’s r ≥ 0.8). The synthetic oversampling technique (SMOTE) was applied to the training set data alone to balance the two classes of lymph nodes, positive and negative [25]. Finally, information gain analysis with a threshold of 0.15 identified the appropriate number of informative parameters to run the ML algorithm in the training set. A support vector machine (SVM) classifier was used for the classification task.

### 2.8. Machine Learning Analysis

**Figure 2:**
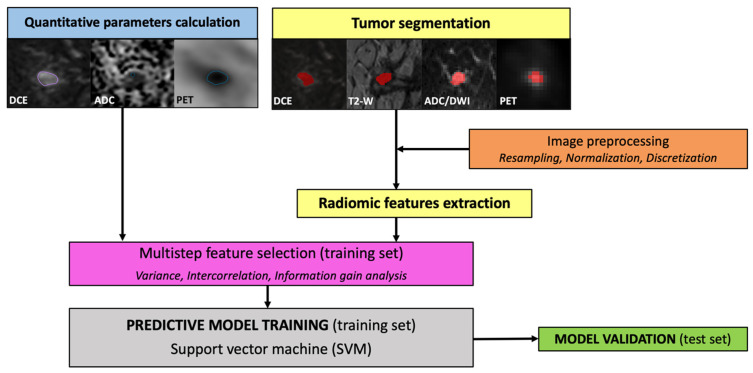
Radiomics pipeline, including image analysis and model development.

A 5-fold stratified cross-validation was used to evaluate the performance of the ML algorithm during the tuning process on the training set. In detail, data are divided into five folds, preserving class balance so that one was used as a validation set for the algorithm trained on the remaining (*n* = 4) data folds. The models are built, and their output is produced to present accuracy metrics and estimate how a similar model would perform on new data. Then, all available training data are used to generate the final model. This model is then tested on new data to confirm inferences made during cross-validation to simulate use in clinical practice on unseen cases. The final model was then applied to the unseen population of the test set. The radiomics and ML pipeline is illustrated in Figure 2.

The performance of the algorithm was also compared to that of two baseline models by comparing the mean accuracy based on 100 iterations of 10-fold cross-validation in the training set, using a corrected paired T-test. The baseline models were a 0 Rules (0R) model, which made inferences based on the class distribution alone (i.e., the mode), and a One Rule (1R) model, which used only the minimum-error feature for prediction. The analysis was performed using the pandas (v1.5.3), imbalanced-learn (v0.10.1), and scikit-learn (v1.2.1) Python packages and the Waikato Environment for Knowledge Analysis data mining platform (v3.9.6).

## 3. Results

### 3.1. Patient and Lesion Characteristics

A total of 117 patients with 127 breast lesions (mean tumor size = 29 mm ± 18 mm) were included in the study, and a lesion-based analysis was conducted. Of the 117 patients, four had bilateral breast lesions, and six had multifocal breast lesions with the same histological and molecular features. Of the 127 breast lesions, 85 were lymph node-positive, and 42 were lymph node-negative. The clinical/histological data of included patients/breast cancer lesions are illustrated in Table 1.

### 3.2. Radiomics and ML Analysis

A total of 4424 radiomics features were extracted per breast lesion (1106 per each PET/MRI sequence) and combined with quantitative parameters (*n* = 5) to determine the final features/parameters to include in the predictive model. As a result of the train/test split, the training set comprised 85 breast lesions (85/127, 67%), of which 57 and 28 were lymph node-positive and negative, respectively. After applying SMOTE to balance between the lymph node-positive negative classes, the training set comprised 114 lesions, with 57 breast lesions in each class. The remaining 42 lesions (85/127, 67%), of which 28 and 14 were lymph node-positive and negative, respectively, were grouped as the test set. Following multi-step feature selection, no low-variant features were found. At intercorrelation analysis, 3848 parameters resulted as highly intercorrelated, leaving 581 features, of which 13 were finally selected by information gain analysis (threshold = 0.15). Of note, only radiomic features were selected, extracted from the log, and wavelet-filtered T2-weighted, DCE, ADC, and PET images (Table 2).

Using these features, the SVM classifier (C = 10, Radial Basis Function kernel) obtained an accuracy of 79.8% (91/114) in discriminating lymph node-positive and negative breast cancer lesions in the training set (area under the curve (AUC) = 0.798, sensitivity = 70.2%, specificity = 89.5%, negative predictive value = 75%, positive predictive value = 87%). An accuracy of 78.6% (33/42) was achieved in the test set (AUC = 0.839, sensitivity = 67.9%, specificity = 100%, negative predictive value = 60.9%, positive predictive value = 100%), which was significantly higher than that of the baseline models of 0R (47.3%) and 1R (63.1%). Confusion matrices of the performance of the SVM classifier in both the training and test set are given in Table 3 and Table 4.

## 4. Discussion

The preoperative, non-invasive prediction of axillary lymph node status in patients with breast cancer is feasible using a radiomics and ML model based on the analysis of primary lesions on simultaneous 18F-FDG PET/MRI. The model developed yielded an accuracy of 79.8% in the training set for the differentiation of lymph node-positive and negative breast cancer. Furthermore, the accuracy remained stable (78.6%) when the model was applied to the test set. This is an important finding regarding the model’s generalizability, encouraging the application of the model to external 18F-FDG PET/MRI datasets. Only radiomic features were retained in the ML model, while quantitative parameters were excluded. This could be because quantitative data on perfusion, diffusion, and metabolism were captured by the radiomic features themselves and resulted, therefore, redundant. The retained radiomics features not only spanned the entire PET/MRI dataset, including both morphological and functional images (i.e., T2-weighted, ADC, DCE, and PET), but also reflected pixel distribution at different complexity levels. T2-weighted, DCE and PET images contributed most and equally to the prediction task. There is growing interest in developing non-invasive tools to predict axillary lymph node status in patients with breast cancer [26,27]. Indeed, metastatic involvement of the axillary lymph nodes strongly affects prognosis and treatment strategies, often shifting patients from surgery to systemic, neoadjuvant treatment [28]. In addition, according to the landmark trials AXOSOG Z0011, IBCSG, and AMAROS, a de-escalating surgical approach (i.e., sentinel lymph node biopsy only) can be proposed to patients with only mild axillary lymph node involvement [29,30,31]. Further, clinical trials are currently ongoing to explore a “non-sentinel lymph node biopsy” option in clinically negative (cN0) patients [11]. If such an approach were to be applied to clinical practice, preoperative imaging would become even more critical to non-invasively and accurately pre-screen for truly node-negative patients. Currently, imaging modalities are still not accurate enough for assessing axillary lymph nodes, with the US being the most sensitive one. However, its accuracy is affected by a variable specificity and its operator dependence, especially when axillary lymph nodes present only mild abnormalities [9,32]. Therefore, accurate and non-invasive imaging-based solutions are desirable, with AI-based approaches being the most promising ones, especially those relying on primary lesion heterogeneity. Most studies applying ML to predict axillary lymph node metastasis have used MRI-derived radiomic features. Two meta-analyses showed that MRI-derived features have a pooled accuracy, sensitivity, and specificity of 89%, 82%, 83%, 80%, 79%, and 77%, respectively [26,27]. However, both meta-analyses were limited by the heterogeneity between studies and the selection of the highest-performing algorithms that could have affected the overall performance results. As such, MRI-derived radiomics still does not meet the requirements for being introduced in clinical practice. Regarding PET images, even fewer preliminary investigations have been conducted for the prediction of axillary lymph node involvement. Chen et al. recently tested different ML algorithms applied to 18F-FDG PET/CT-derived radiomics and non-radiomics features of primary breast lesions for the detection of axillary metastasis in clinically node-negative breast cancer [33], with the random forest classifier showing the best AUC values (0.661–0.929). Similarly, Song applied an XGBoost algorithm to VOIs of PET images to predict nodal status in 100 invasive ductal carcinomas, obtaining sensitivity, specificity, and accuracy of 90.9%, 71.4%, and 80%, respectively, in the test set [34].

ML was also recently applied to clinically assessable 18F-FDG PET/MRI features of axillary lymph nodes, such as the presence/absence of fatty hilum, contrast enhancement, and margins, for the prediction of N status, showing a performance similar to that of expert readers (diagnostic accuracy of 91.2% vs. 89.3%, respectively) [35].

To the best of our knowledge, only one previous study has assessed the feasibility and usefulness of an axillary status predictive model using ML and radiomics features extracted from simultaneous 18F-FDG PET/MRI. In detail, the authors used a patient-based analysis and internal 5-fold cross-validation. The obtained diagnostic performance was similar to that of our investigation (AUC = 0.810, 95% CI: 0.740–0.881) [36]. Innovative aspects of our approach compared to this previous study include the operator independence of the selected radiomic features and the use of a test set to validate the developed model.

Our investigation presents several limitations, the first being the small sample size, mainly due to the limited access to simultaneous 18F-FDG PET/MRI. However, it was still possible to divide the sample into a training and a test set to preliminarily assess the generalizability of the developed model. Nevertheless, the validation of this model on an external population would be beneficial. A further limitation is related to the heterogeneity of histological breast cancer phenotypes. However, a predictive model meant to work in routine clinical practice should be able to perform the prediction task independently from tumor histological features, which may be unknown at the time of 18F-FDG PET/MRI examination. Finally, our investigation did not include a comparison with the highest-performing imaging modality in clinical practice, i.e., the US. Indeed, a fair comparison was not possible as US examinations were not prospectively performed along with PET/MRI examinations. A retrospective analysis could have been performed based on clinical reports, but these were not available in all cases and were also produced by several operators with different levels of experience. Such a comparison would also not strengthen the results of our study. Additionally, a comparison between AI and human operators reading PET/MRI images was also not feasible, as axillary regions were not properly covered in all sequences of the dedicated breast MRI protocol. Based on our findings and experience, prospective validation of the ML algorithm in clinical practice, including conventional imaging assessment with and without the availability of ML scores, would be advisable to fully assess its clinical value.

## 5. Conclusions

An ML approach using simultaneous 18F-FDG PET/MRI-derived radiomic features proved feasible and accurate for predicting axillary lymph node metastasis in patients with breast cancer. The possibility of implementing this model into clinical practice may significantly improve treatment planning while addressing patients to systemic treatment or de-escalating surgery and providing a comprehensive PET/MRI tool for breast cancer local and N staging.

## Figures and Tables

**Table 1:** Histological features of included breast lesions.

Histological Type	Number of Lesions	%
IDC	101	79
ILC	12	9
IDC + ILC	5	4
Metaplastic carcinoma	1	1
Mucinous carcinoma	2	2
Papillary carcinoma	4	3
Tubular carcinoma	1	1
Apocrine carcinoma	1	1
Total	127	100
**Molecular subtype**	**Number of lesions**	**%**
Luminal A	12	9
Luminal B	69	54
HER2+	14	11
Triple-negative	32	26
Total	127	100
**Tumor grade**	**Number of lesions**	**%**
G1	12	9
G2	47	37
G3	66	52
Not available	2	2
Total	127	100

**Abbreviations:** IDC, invasive ductal carcinoma; ILC, invasive lobular carcinoma; HER2, human epidermal growth factor receptor 2.

**Table 2:** Selected radiomics features.

T2_wavelet-LHH_ngtdm_Complexity
T2_wavelet-LLL_firstorder_Minimum
T2_wavelet-HLH_firstorder_Kurtosis
T2_log-sigma-1-0-mm-3D_ngtdm_Complexity
ADC_wavelet-LHL_gldm_LargeDependenceLowGrayLevelEmphasis
DCE_wavelet-LHH_glcm_ClusterProminence
DCE_wavelet-LHL_firstorder_RootMeanSquare d
DCE_wavelet-HHL_firstorder_Median
DCE_wavelet-HHL_firstorder_Maximum
PET_log-sigma-3-0-mm-3D_glrlm_GrayLevelVariance
PET_wavelet-LHH_glcm_Autocorrelation
PET_wavelet-LHH_glszm_SmallAreaHighGrayLevelEmphasis
PET_wavelet-LHL_glszm_SmallAreaHighGrayLevelEmphasis

**Table 3:** Confusion matrix of the support vector machine classifier in the training set (*n* = 114).

SVM Classification	Ground Truth		Total
	Lymph Node-Positive	Lymph Node-Negative	
Lymph node-positive	40	6	46
Lymph node-negative	17	51	68
**Total**	57	57	114

Abbreviations: SVM, support vector machine.

**Table 4:** Confusion matrix of the support vector machine classifier in the test set (*n* = 42).

SVM Classification	Ground Truth		Total
	Lymph Node-Positive	Lymph Node-Negative	
Lymph node-positive	19	0	19
Lymph node-negative	9	14	23
**Total**	28	14	42

Abbreviations: SVM, support vector machine.

## Data Availability

Research data are available from the Corresponding Author by reasonable request.

## Supplementary Materials

The following supporting information can be downloaded at https://www.mdpi.com/article/10.3390/cancers15205088/s1, Supplementary S1. 18F-FDG PET/MRI acquisition protocol; Table S1. Details of the breast MRI acquisition protocol; Supplementary S2. Perfusion parameters measurements.
