# Supplementary material for: Simultaneous 18F-FDG PET/MRI Radiomics and Machine Learning Analysis of the Primary Breast Tumor for the Preoperative Prediction of Axillary Lymph Node Status in Breast Cancer
Source: Cancers (Basel). 2023 Oct 21;15(20):5088. doi: 10.3390/cancers15205088 (PMC10604950; doi:10.3390/cancers15205088)
Supplement: Supplementary file 1 [file cancers-15-05088-s001.zip › cancers-2584983-supplementary.pdf]

Supplementary Material

Supplementary S1. ^{18}F-FDG PET/MRI acquisition protocol

Patients were required to fast for five hours prior to the imaging examination, to ensure blood glucose levels < 150 mg/mg/dL. Body weight-adapted (200–350 MBq/kg body weight) ^{18}F-FDG was then intravenously injected. After an uptake time of 60 min, PET/MRI acquisition started. Attenuation correction was performed using the standard Dixon-based attenuation correction method. A 3D acquisition technique was employed with an axial and transverse field of view (FOV) of 26 and 59 cm, respectively, and a sensitivity of 13.2 cps/kBq. Ordinary Poisson 3D ordered subset expectation maximization (OP-OSEM) (with Gaussian scatter correction) was used for the reconstruction of static PET images using 3 iterations and 21 subsets to produce a 172-image matrix 1.0 zoom including all standard corrections (normalization, scatter, random coincidences, and decay).

The acquisition of PET data lasted 30 minutes. MRI of the breast was performed using a dedicated 16-channel breast coil (Rapid Biomedical, Germany), with the following sequences: axial T2-weighted sequence; axial diffusion tensor imaging (DTI) single-shot spin-echo-prepared echo-planar imaging (EPI) sequence with parallel imaging and fat suppression, twelve directions, and two b-values (0 and 800); and high temporal resolution (16.7 s) T1-weighted Time-resolved angiography WIth Stochastic Trajectories (TWIST) DCE sequence, preceded by five pre-contrast gradient echo sequences with variable flip angles (2°, 10°, 20°, 30°, and 40°). Following an update to the breast MRI protocol (December 2017), high temporal resolution (14 s) T1-weighted TWIST Dixon dynamic sequence with 23 measurements was acquired as DCE sequence. DCE images were obtained before and after intravenous injection of macrocycle-structured gadolinium-based contrast agent (Gadoteric acid Dotarem; Guerbet, Aulnay-sous-Bois, France) at a dose of 0.2 ml/kg body weight and a flow rate of 3.5 ml/s. Details of the breast MRI protocol are reported in **Table S1**.

Table S1. Details of the breast MRI acquisition protocol.

Sequence	Acquisition plane	TR (ms)	TE (ms)	Matrix size	FOV (mm)	Slice thickness (mm)	Gap (mm)	Flip angle
**T2-weighted**	Axial	4820	192	640×480	360×360	2.5	3	128°
**DTI**	Axial	4500	87	190×112	212×360	4	5.2	90°
**DCE – TWIST**	Axial	4.7	2.46	448×448	340×340	2	0	15°
**DCE – Dixon***	Axial	4.7	1.3	352×352	440×440	2	0	10.5°

Abbreviations: DTI, diffusion tensor imaging; DCE, dynamic contrast-enhanced; TWIST, Time-resolved angiography WIth Stochastic Trajectories

*After MRI update

Supplementary S2. Perfusion parameters measurements.

The arterial input function was estimated by placing 2D ROIs at the level of the right ventricle to generate multiparametric perfusion maps to calculate the following perfusion parameters: mean transit time (MTT), related to the speed of blood in the capillary within the area of interest; volume of distribution (VD), related to the concentration of the contrast agent; and plasma flow (PF), related to the amount of plasma that perfuses the tissue per unit time. Of note, to extract perfusion parameters, the readers contoured the lesions on early post-contrast subtracted images, which better represent tumor aggressiveness, with 2D ROIs drawn over the enhancing tumor tissue and excluding macroscopic areas of necrosis, cystic content, or intra/perilesional blood vessels. The 2D ROIs were then matched with corresponding perfusion maps, and MTT, PF, and VD mean values were calculated and recorded.
